# Design and Testing of a Smart Facemask for Respiratory Monitoring during Cycling Exercise

**DOI:** 10.3390/bios13030369

**Published:** 2023-03-10

**Authors:** Chiara Romano, Andrea Nicolò, Lorenzo Innocenti, Massimo Sacchetti, Emiliano Schena, Carlo Massaroni

**Affiliations:** 1 The Departmental Faculty of Engineering, Università Campus Bio-Medico di Roma, 00128 Rome, Italy; 2 Department of Movement, Human and Health Sciences, University of Rome “Foro Italico”, 00135 Rome, Italy

**Keywords:** wearable sensors, validity, respiratory frequency, cadence, measurement accuracy, exercise, sport, breathing, respiratory rate

## Abstract

Given the importance of respiratory frequency (*f*_R_) as a valid marker of physical effort, there is a growing interest in developing wearable devices measuring *f*_R_ in applied exercise settings. Biosensors measuring chest wall movements are attracting attention as they can be integrated into textiles, but their susceptibility to motion artefacts may limit their use in some sporting activities. Hence, there is a need to exploit sensors with signals minimally affected by motion artefacts. We present the design and testing of a smart facemask embedding a temperature biosensor for *f*_R_ monitoring during cycling exercise. After laboratory bench tests, the proposed solution was tested on cyclists during a ramp incremental frequency test (RIFT) and high-intensity interval training (HIIT), both indoors and outdoors. A reference flowmeter was used to validate the *f*_R_ extracted from the temperature respiratory signal. The smart facemask showed good performance, both at a breath-by-breath level (MAPE = 2.56% and 1.64% during RIFT and HIIT, respectively) and on 30 s average *f*_R_ values (MAPE = 0.37% and 0.23% during RIFT and HIIT, respectively). Both accuracy and precision (MOD ± LOAs) were generally superior to those of other devices validated during exercise. These findings have important implications for exercise testing and management in different populations.

## 1. Introduction

Ever-growing technological development is offering new solutions to monitor athletes and exercise practitioners with wearable sensors in applied settings. The physiological and mechanical variables currently available may help refine the prescription and monitoring of training and assess its effects, with potential benefits for the health and exercise performance of different populations. One of the physiological variables that is gaining particular interest in the exercise community is respiratory frequency (*f*_R_) [1,2], also considering the numerous techniques suitable for its measure [3,4]. Unlike other physiological variables (including tidal volume, V_T_), *f*_R_ is closely associated with perceived exertion [5,6,7,8,9], and its time course reflects changes in exercise tolerance [10]. The fact that the depth (i.e., V_T_) and rate (i.e., *f*_R_) of breathing provide different information is supported by their differential control, as V_T_ and *f*_R_ are mainly modulated by metabolic and non-metabolic inputs, respectively [8,10,11,12]. As such, *f*_R_ has been defined as the behavioral component of minute ventilation [1,10].

The notion that *f*_R_ is a valid marker of physical effort, especially at high intensities, is particularly evident during cycling exercise and applies to a variety of exercise paradigms, including incremental exercise [13], time trial [6,8,14], time to exhaustion [5], trapezoidal exercise [11], all-out exercise [15], and high-intensity interval training (HIIT) [8,14,16]. HIIT shows a feature of *f*_R_ that distinguishes the behavioral component of minute ventilation from commonly monitored physiological variables such as oxygen uptake, heart rate, and blood lactate. Indeed, *f*_R_ responds rapidly to the alternation of work and recovery, while the other variables show a delayed response both at the onset and offset of the work phase [8,16,17,18]. This offers an advantage in monitoring cycling exercise as well as other sports that are characterized by intermittent activities [1,7].

While the measurement of *f*_R_ and other ventilatory variables has a long tradition in exercise physiology laboratories, interest in custom-made and commercial wearable devices suitable for *f*_R_ monitoring in applied exercise settings has increased substantially in recent years [1,2]. Different methods can be used to monitor *f*_R_ in applied exercise settings, and some of them are attracting particular attention, including the techniques recording chest wall movements and those extracting *f*_R_ from physiological signals modulated by breathing (e.g., the electrocardiographic signal). Among the sensors recording chest wall movements, those measuring strain can be easily integrated into clothes or textiles, which facilitates the development of unobtrusive devices. Different wearables integrating strain sensors have been developed and tested during exercise, generally showing a relatively good performance in measuring *f*_R_ [19,20,21,22,23,24,25,26,27]. The other emerging trend in the field of exercise is the attempt to extract *f*_R_ from cardiac signals registered from heart rate straps or other devices, where the main advantage is that these signals are already recorded by many athletes and exercise practitioners [28,29]. However, the error in estimating *f*_R_ from cardiac signals is usually higher than that found when strain sensors are used [28,29]. A common problem of the aforementioned techniques is that the registered respiratory signal is susceptible to motion artefacts, which implies that the evaluation of the validity of *f*_R_ measurement should be sport specific. Indeed, motion artefacts may affect the quality of the respiratory signal more in some sports (e.g., running) than others (e.g., cycling). However, even in cycling, some exercise paradigms may challenge the quality of the respiratory signal. For instance, we found that the error in measuring *f*_R_ was higher during HIIT compared to incremental exercise, even when the signal was recorded with a differential pressure sensor and was thus minimally affected by motion artefacts [30]. Hence, the use of HIIT in validation studies is encouraged but currently underappreciated. Another important overlooked requirement of validation studies is the need to assess the quality of the respiratory signal at a breath-by-breath level because different respiratory services may need such a level of detail (e.g., real-time monitoring) [1,30]. The quality of validation studies performed during exercise should improve accordingly.

The variety of sensors available for measuring *f*_R_ allows for the identification of other wearable solutions that have so far been considered to a limited extent and may partially solve some of the above-mentioned problems. We have recently started exploiting the very good metrological characteristics of some temperature sensors recording a respiratory waveform that is minimally affected by motion artefacts [31]. This feature of temperature sensors has long been appreciated in the field of exercise physiology, where thermistors or thermocouples have been used for the assessment of the locomotor-respiratory coupling phenomenon [32,33,34,35,36,37], which requires a very good signal quality for its detection. However, the characteristics of temperature sensors have rarely been exploited for the development of wearable sensors suitable for exercise monitoring, which is currently facilitated by the miniaturization of electronics. In a first attempt, we located a thermistor embedded into a small electronic board close to the orifice of a face mask used for laboratory exercise testing, and we found relatively low errors in measuring *f*_R_ [31]. However, this preliminary solution was still far from the usability requirements needed for exercise monitoring in applied settings. Hence, in this study, we present the design and testing of a new solution exploiting the use of a temperature biosensor located in a facemask for *f*_R_ monitoring during cycling exercise.

## 2. Smart Facemask: Design and Description

The smart device presented in this paper has been designed from both a hardware and software perspective to meet the requirements of *f*_R_ monitoring during cycling exercise without restricting the athlete’s mobility or influencing the proper execution of exercise sessions. Hence, in the design and development of the smart device, we considered the following requirements: (i) keep the weight and dimensions as low as possible; (ii) guarantee the washability of components in contact with the athlete after use, to have the correct level of hygiene; (iii) guarantee the continuous data acquisition for more than 10 h, which is a duration that largely exceeds the usual requirements of elite cyclists [38]; (iv) ensure low air resistance to the athlete’s respiratory flow; (v) ensure low motion artefacts sensitivity.

**Figure 1:**
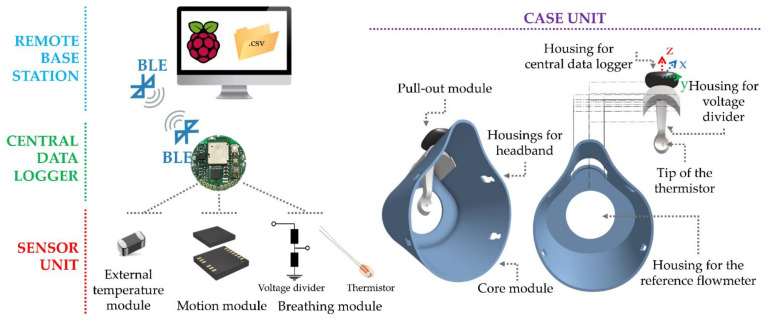
Schematization of the system architecture with the main units (i.e., remote base station, central data logger, sensor unit, and case unit) and their modules.

To fulfil all the requirements, the system’s design was intended to avoid any sensing element in contact with the subject and to maximize the sensitivity to respiratory activity while minimizing the influence of motion artefacts. We decided to develop a smart facemask with a modular architecture that consists of four main units: (i) *central data logger*: designed for data acquisition, digitalization, and transmission; (ii) *remote base station*: designed for sending data messages to the central data logger and to store the raw sensor data; (iii) *sensor unit*: designed to be the sensing part of the whole system; (iv) *case unit*: designed to fit the subject’s face and to integrate both the central data logger and the sensor unit. An example of all the main units and their modules is shown in Figure 1.

The characteristics of the four main units of our system are as follows:

(1) The *sensor unit* includes three modules: (i) a *breathing module* that uses an NTC Radial Glass Thermistor (model number: G10K3976B1, TE connectivity, Resistance and Tolerance @ +25 °C: 10 kΩ and 1%; time response in stirred oil: 0.4 s; Beta Value 25/85: 3976 K) and a voltage divider with a fixed resistance of 10 kΩ. The glass body of the selected thermistor provides a hermetic seal and voltage insulation; hence it can operate in high temperature and moisture environments. This module was designed to monitor respiratory activity by collecting the temperature of the airflow exhaled by the nose and mouth (i.e., T_Exp_); (ii) a *motion module* that consists of a triaxial accelerometer (model Bosch^®^ BMI160) with ±16 g scale. This module was added to provide an estimation of exercise-induced head accelerations that may provide information on the athlete’s movement (e.g., pedalling cadence); (iii) an *external temperature module* that consists of a surface-mount temperature sensor (model number NCP15XH103F03RC, accuracy ±5 °C) positioned so as not to be hit by the flow of air exhaled by the subject. This module was selected to collect the external temperature (i.e., system’s working temperature, henceforth T_E_).

(2) The *central data logger* consists of a small (diameter: 24 mm, height: 6 mm) and low-weight (56.7 g) electronic board that integrates a Bluetooth low energy (BLE) module (Bluetooth LE 4.2–2.4 GHz), the motion module and the external temperature module. In addition, it has four general-purpose I/O (GPIO) ports, a power supply port (3 V), and a 10-bit analog to digital converter (ADC). One of the GPIOs houses the breathing module that is also supplied by the power supply port. The analog-acquired signal is then sent to a 10-bit ADC in the acquisition board for digitalization. This module allows us to receive data messages from the remote base station. The central data logger is supplied by an interchangeable lithium coin cell (capacity up to 240 mA/h, 20–54 °C) that guarantees more than 700 h of continuous acquisition, widely sufficient for a typical training session.

(3) The *remote base station* consists of a Raspberry Pi that allows sending data messages in a Python environment to the central data logger to provide instructions to (i) set the sampling frequency of each sensor unit (i.e., 50 Hz for the breathing and motion modules; 1 Hz for the external temperature module) and start the data recording inside the central data logger internal memory (8 MB); (ii) stop the data acquisition; (iii) begin the data download; (iv) retry the connection during the download in the case of connection drops. Additionally, it allows us to store the raw sensor data in a csv format when sent from the central data logger via BLE.

(4) The *case unit* was designed with two detachable modules. The first module is called the *pull-out module* and is made up of a housing for the sensor unit and a housing for the central data logger, as shown in Figure 1. This is made up of 3D-printed polylactic acid with a Creality Ender-3 v2 in the shape of a ‘spoon’ with an overall weight of 7 g. The design was intended so that most of the nose and mouth airflow hits the tip of the thermistor (approximately 2 cm away from the tip of the mouth) of the breathing module, which emerges from the concave side of the spoon (see Figure 1). This configuration makes it possible to record temperature changes during the inspiratory and expiratory phases of breathing. On the other hand, the ‘spoon’ was specifically designed to make the thermistor unexposed to wind. At the top of the ‘spoon’, we designed housing for the central data logger positioned in the central part of the subject’s head to obtain a more reliable signal of the head’s accelerations from the motion module. The second module of the case unit is called the *core module* and was realized to be completely electronics-free. It is a facemask made from 3D-printed Thermoplastic Polyurethane 95A, with an overall weight of 45 g. This module was designed to accommodate the pull-out module so that the latter can be hooked during the test and unhooked when the facemask is washed and sanitized. It also has a 40 mm diameter hole in the front to ensure low air resistance to the athlete’s airflow. This prevents the proposed mask from forming a closed space around the nose and mouth once fitted. Finally, it has four housings for the headband that can be worn and set by the athlete so that the facemask properly fits the face.

In this paper, we will focus mainly on the breathing module of the sensor unit (see Figure 1) and its performance. The system’s working principle, the developed algorithm for respiratory analysis, preliminary tests, and the validation process conducted against a reference flowmeter on athletes are presented below.

## 3. System’s Principle of Working and *f*_R_ Estimation: Algorithm Design

**Figure 2:**
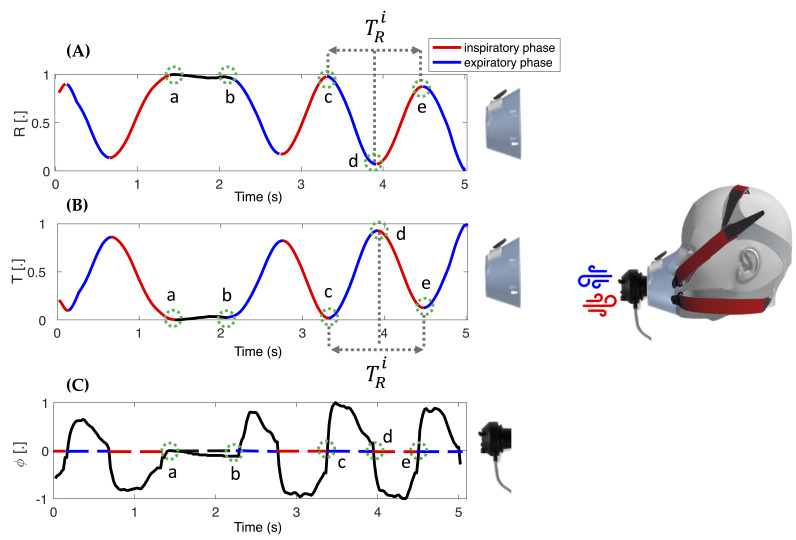
Example of a normalized resistance signal (**A**) and the resulting normalized temperature signal (**B**) collected during breathing from the smart facemask. Black line (a–b) is the end-inspiratory apnea; blue line (c–d) is the expiratory phase; red (d–e) is the inhalation phase. In (**C**), the normalized signal was collected during breathing from the reference flowmeter used in the validation phase. The black dashed line (a–b) is the end-inspiratory apnea; the blue dotted line (c–d) is the expiratory phase, and the red dotted one (d–e) is the inhalation phase.

The operating principle of our system for collecting respiratory biosignals is based on variations in the air temperature flowing out of the nose and mouth during the breathing activity to which it is exposed. Our sensor has a variable resistance (R) as the temperature (T) varies with a negative transformation coefficient. Therefore, when the T increases, the R decreases. In the absence of exhaled airflow (e.g., sensor not worn by the subject or during apnea), the respiratory module is surrounded by T_E_, so the output temperature signal (and the resistance signal) remains stable. On the other hand, during exhalation, the airflow impinges on the respiratory module at a temperature (T_Exp_) of approximately 37 °C [39]. Thus, the behavior of the biosensor is as follows:

- If T_Exp_ > T_E_, the temperature measured by the respiratory module increases, and its resistance decreases (see c–d in Figure 2);
- If T_Exp_ < T_E_, the temperature measured by the respiratory module decreases, and its resistance increases;
- If T_Exp_ = T_E_, the temperature (and the resistance) measured by the respiratory module remains stable (see a–b in Figure 2).

Instead, in the inhalation phase, the respiratory module sensor is re-exposed to T_E_. Therefore, the phenomenon is ‘passive’ and behaves as follows:

- If T_Exp_ > T_E_, the temperature measured by the respiratory module decreases, and its resistance increases (see d–e in Figure 2);
- If T_Exp_ < T_E_, the temperature measured by the respiratory module increases, and its resistance decreases.

**Figure 3:**
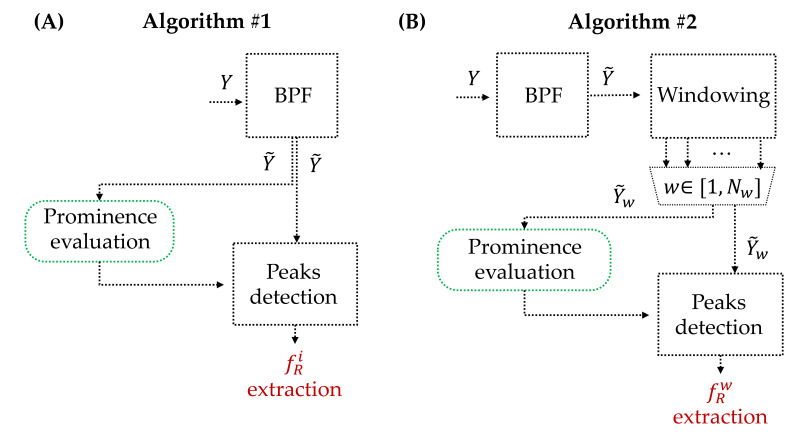
(**A**) Block diagram of Algorithm#1 for extracting the breath-by-breath respiratory frequency from the respiratory signal; (**B**) block diagram of Algorithm#2 for extracting the average respiratory frequency in 30 s windows of the respiratory signal. BPF: band-pass filter; $f_{R}^{i}$: *i*-th respiratory frequency; $f_{R}^{w}$: mean respiratory frequency in the *w*-th window.

To extract *f*_R_ from the collected respiratory signals Y, two different algorithms were implemented, as shown in Figure 3. The first algorithm was used to extract the breath-by-breath *f*_R_, while the second algorithm was used to extract the average *f*_R_ in 30 s windows. In both algorithms, the breathing signal was filtered with a Butterworth band-pass filter (BPF) with cut frequencies of 0.01 Hz and 2 Hz to obtain $\tilde{Y}$ [1,40,41]. Hence, the components of physiological breathing frequency values are preserved in the signal.

Subsequently, with Algorithm#1, the *f*_R_ was directly extracted from the $\tilde{Y}$. For this purpose, the peaks corresponding to the end of exhalation were selected with an algorithm based on both temporal and amplitude criteria to exclude artefacts. The temporal criterion states that two consecutive peaks are selected as separate events if their distance exceeds a minimum value set at 0.6 s because, even during maximal effort exercise, the *f*_R_ of human adults usually remains below 1.67 Hz (100 breaths/min) except for extreme exercise conditions [15]. Regarding the amplitude criterion, we set a percentage threshold of 2% of the maximum peak-to-peak amplitude of the signal in order to be robust to changes in signal amplitude. Only peaks exceeding this threshold were selected as breathing events, while the others were discarded. The distance between two different breathing cycles ($T_{R}^{i}$) was calculated as the elapsed time between two consecutive breathing events. Consequently, the breath-by-breath *f*_R_ (i.e., $f_{R}^{i}$) was calculated as 60/$T_{R}^{i}$.

Algorithm#2 employs the previously described algorithm (Algorithm#1) within 30 s windows. Thus, in each window, the breath-by-breath *f*_R_ (i.e., $f_{R}^{i,w}$) was computed, and the average one was calculated (e.g., $f_{R}^{w}$ in the w-th window). This resulted in an average $f_{R}^{w}$ value for each window.

## 4. In-Lab Tests with the Mechanical Ventilator: Description and Results

**Figure 4:**
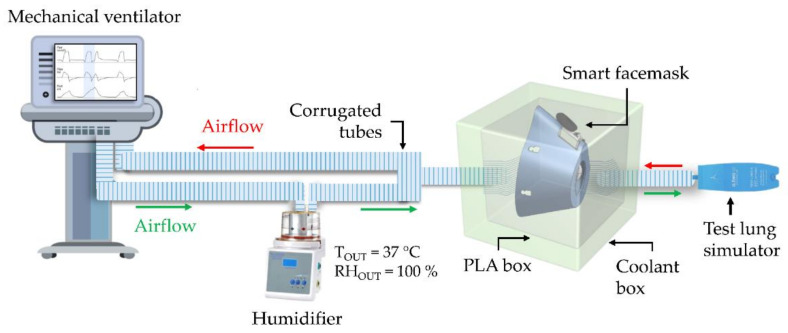
Schematization of the experimental setup for preliminary in-lab tests with the mechanical ventilator.

To assess the feasibility of the smart facemask in monitoring *f*_R_, preliminary tests were conducted in the laboratory. The aim of this preliminary analysis is to investigate the behavior of the entire system when it is provided with simulated respiratory flows at set frequencies ranging from 5 to 75 bpm. For this purpose, we used an experimental setup consisting of (i) a mechanical ventilator (SERVO VENTILATOR 300, SIEMENS, *f*_R_ accuracy ±1 bpm or 10% of the set value) used to provide frequency-controlled air flows; (ii) a humidifier (MR850ALU, Fisher&Paykel HEALTHCARE) used to heat and humidify the air coming out of the mechanical ventilator; (iii) a 3D-printed Polylactic Acid PLA box containing the smart facemask. This box is designed with a front hole to convey the airflow onto the breathing module and a rear hole that allows the airflow to pass through, minimizing losses; (iv) a coolant box that aims to keep the T_E_ outside the facemask almost constant at ~25 °C; (v) a lung simulator that aims to simulate the adult’s respiratory airways. Thus, the airflow originating from the mechanical ventilator passes through the humidifier to be heated and humidified (temperature 37 °C, relative humidity 100%). The heated and humidified flow is directed into the PLA box through a corrugated tube connected to the front hole and impinges on the thermistor of the respiratory module of the smart facemask. Subsequently, the flow exits through the rear hole and reaches the test lung simulator, which captures the flow and then returns it to the mechanical ventilator. An example of the whole system is shown in Figure 4.

Experiments were conducted to simulate eight different *f*_R_ levels. The set *f*_R_ ranged from 5 bpm to 75 bpm in steps of 10 bpm to investigate the typical frequencies of a subject at rest and during exercise [1]. For each set *f*_R_, 10 respiratory acts were simulated.

**Figure 5:**
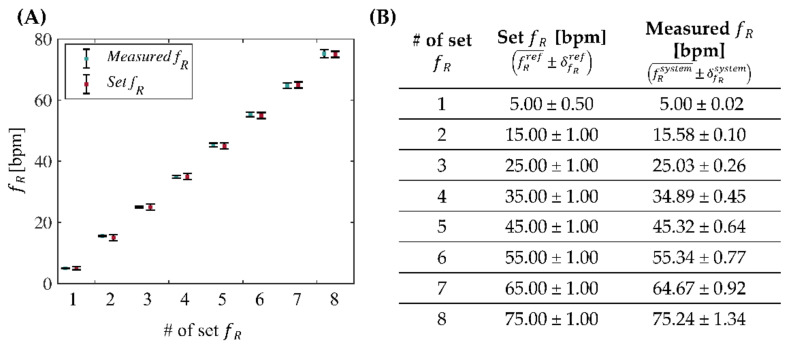
Mean and uncertainty of the values measured with the smart facemask (in blue) and the values set with the mechanical ventilator (in red) (**A**) and related table (**B**).

To evaluate the performance of the developed device, the breath-by-breath *f*_R_ was extracted from the respiratory module signal with Algorithm#1 and compared to the frequency values set on the mechanical ventilator. For each set *f*_R_, the measured values were reported as shown in Equation (1):

(1) $$f_{R}{}_{mis}=\bar{f_{R}}\pm \delta_{f_{R}},\;$$

where $\bar{f_{R}}$ denotes the mean of the measurements and $\delta_{f_{R}}$ is the uncertainty calculated using Student’s distribution and expressed as follows in Equation (2):

(2) $$\delta_{f_{R}}=k\frac{S_{f_{R}}}{\sqrt{N}}\;,$$

where *k* is equal to 2.306 and denotes the coverage factor considering a 95% confidence level, $S_{f_{R}}$ is the standard deviation of the measurements, and *N* is the number of the extracted *f*_R_ values (i.e., 9). The deviation obtained by comparing the values measured with the developed system and those set on the mechanical ventilator is shown in Figure 5.

We examined the discrepancy between the measurements provided by the two instruments, defined as the difference between two measured values of the same quantity with both the device and the reference system. Hence, we assessed whether there were any points in common between the interval $\left|\bar{f_{R}^{system}}-\delta_{f_{R}}^{system},\bar{f_{R}^{system}}+\delta_{f_{R}}^{system}\;\right|$ and the interval $\left|\bar{f_{R}^{ref}}-\delta_{f_{R}}^{ref},\bar{f_{R}^{ref}}+\delta_{f_{R}}^{ref}\;\right|$. Figure 5 shows that there is no discrepancy between the two measurements for all the tested *f*_R_. Additionally, the results exhibit an average error of less than 0.6 bpm with a maximum deviation of the mean values of 0.58 bpm for a set frequency of 15 bpm and an overall overestimation of the proposed system with respect to the reference in all but two set frequencies (i.e., 35 and 65 bpm).

## 5. Validation of the Smart Facemask and Algorithms: Experimental Tests on Athletes

Experimental tests were carried out to validate the proposed smart facemask against a reference instrument for *f*_R_ monitoring and pedalling cadence estimation.

(1) *Population, Experimental Setup, and Protocol:* Ten trained healthy non-smoker cyclists (10 males, mean ± SD: age 25 ± 6 years, body mass 68 ± 6 kg, height 174 ± 5 cm) were enrolled for this purpose. The study was approved by the Institutional Review Board of the University of Rome “Foro Italico” (CAR 112/2021). The principles of the Declaration of Helsinki were followed in all steps of the study, and written informed consent for study participation was signed by all volunteers. To validate the proposed device, we conducted an indoor testing session at T_E_ of about 26 °C (obtained by averaging all the recorded T_E_ during the indoor tests). A gold-standard flowmeter (Quark PFT, COSMED S.r.l., Rome, Italy) was used to collect reference respiratory airflow (ϕ) at 50 Hz.

**Figure 6:**
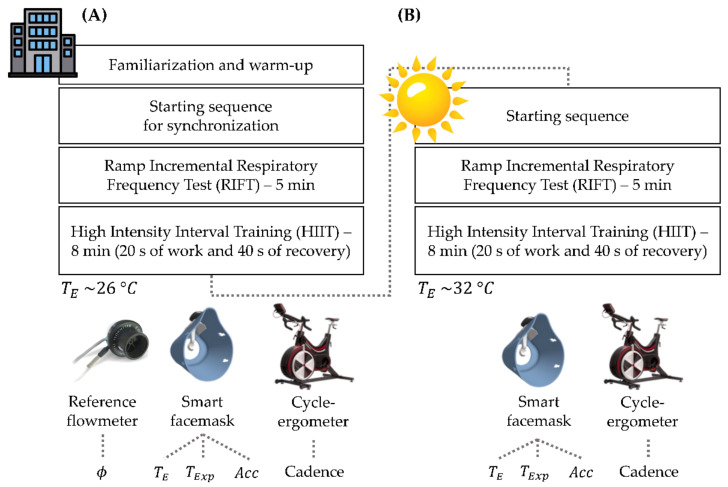
Description of the experimental protocol carried out during the (**A**) indoor testing session for the validation of the smart facemask for respiratory frequency monitoring (at average T_E_ = 26 °C) and (**B**) the outdoor testing session (at average T_E_ = 32 °C), and schematic representation of the devices used.

Before starting the test, each volunteer was asked to wear the developed smart facemask with a properly adjusted headband to keep it fixed on the face. The flowmeter was placed in a dedicated hole realized on the core module of the smart facemask. The test was performed on a cycle-ergometer (WattBike Pro, model WAT-1W51-015-15) that was set for each participant based on comfort and anthropometric characteristics. After an initial familiarization phase and a 3 min warm-up, participants were encouraged to perform a starting sequence consisting of three deep breaths followed by apnea. This procedure was used to synchronize the respiratory signals collected with the smart facemask and the reference system. Then, they were asked to complete two different exercise protocols interspersed by 3 min of recovery (see Figure 6A):

1. Ramp incremental respiratory frequency test (henceforth referred to as RIFT), where participants were asked to replace spontaneous breathing with the *f*_R_ paced by a metronome. This test lasted 5 min, and the *f*_R_ paced by the metronome increased from 20 bpm to 75 bpm in an exponential fashion. Participants were asked to cycle during this test and to self-select pedalling cadence and power output according to preference. The execution of this test has several advantages: (i) the time course of *f*_R_ resembles the response commonly observed during incremental exercise [10,42]; (ii) the range of *f*_R_ values exceeds the range of values commonly observed during cycling exercise [1,6,14]; (iii) it allows for the evaluation of the quality of the respiratory signal even when no reference system is used (see Section 6 Outdoor Tests).
2. High-intensity interval training (henceforth referred to as HIIT) test composed of eight repetitions of 20 s of work and 40 s of recovery. The work-phase power output was self-selected by the cyclist to reach approximately a value of 19 of the Borg’s 6–20 ratings of perceived exertion scale [43] on the last of the eight repetitions.

### 5.1. Breathing Module for f_R_ Monitoring

Prior to data analysis, all the signals recorded with the facemask (T_Exp_ signal, T_E_ signal, motion module signal) were synchronized with the *ϕ* reference signal starting from the third deep breath of the starting sequence. The reference *ϕ* provides a pseudo-periodic signal with a negative phase corresponding to inspiration and a positive phase corresponding to expiration (see Figure 2C); *ϕ* signal was integrated to obtain a volume signal (V), in accordance with previous studies [44,45]. V has an increasing trend during exhalation and a decreasing trend during inhalation, comparable to the signal acquired with our device. Algorithm#1 and Algorithm#2 were applied to T_Exp_ and V to estimate the $f_{R}$ (breath-by-breath $f_{R}$) and $f_{R}^{W}$ (average $f_{R}$ in 30 s-windows), respectively.

For both algorithms, comparison between data provided by the developed system and those from the reference device was performed using the Bland-Altman analysis [46]. The latter was carried out to assess bias between the two measurement systems and expressed in terms of the mean of differences (MOD) and limits of agreement (LOAs), calculated as 1.96 times the standard deviation of the differences between the $f_{R}$ values estimated by the two methods.

In addition, the following statistical indexes were calculated: the mean absolute error (MAE) and the mean absolute percentage error (MAPE), respectively, defined as in Equations (3) and (4):

(3) $$MAE\;\left[bpm\right]=\frac{1}{N}\overset{N}{\underset{j=1}{\sum }}\left|f_{R\;device}^{j}-f_{R\;reference}^{j}\right|,$$

(4) $$MAPE\;\left[\%\right]=\frac{1}{N}\overset{N}{\underset{j=1}{\sum }}\left|\frac{f_{R\;device}^{j}-f_{R\;reference}^{j}}{f_{R\;device}^{j}}\right|*100,$$

where *N* represents the number of breaths extracted or the number of windows when using Algorithm#1 or Algorithm#2, respectively.

Table 1A,B shows the number of breaths extracted using Algorithm#1, the MOD ± LOAs from the Bland–Altman analysis, and the MAE and MAPE values calculated for the RIFT test and HIIT test, respectively. Table 2A,B shows the results of the 30 s-window analysis extracted using Algorithm#2 and the number of windows in which the signals were windowed.

**Figure 7:**
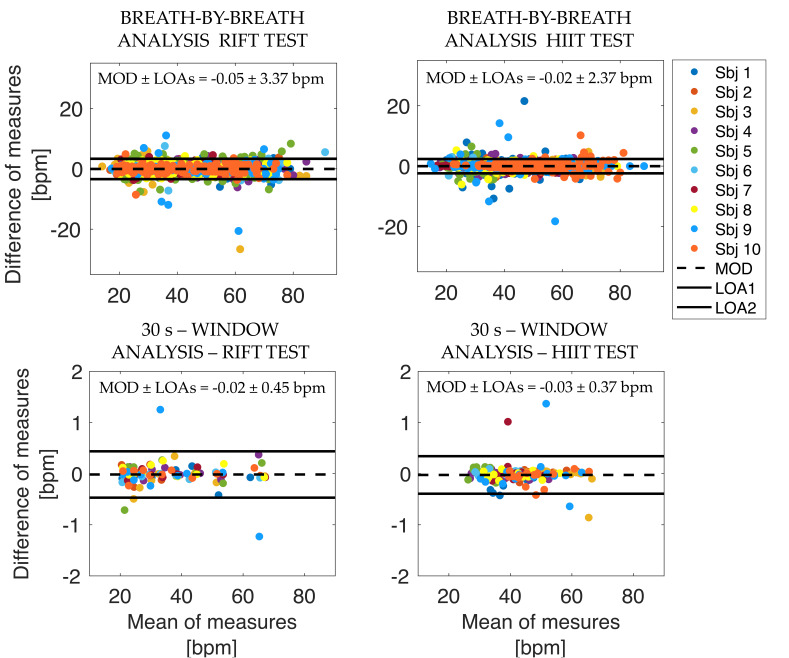
Bland–Altman graphs for both the RIFT test and the HIIT test.

For the sake of clarity, the graphs of the Bland–Altman analysis performed for both the HIIT and RIFT test by all subjects in the indoor test are shown below (see Figure 7).

### 5.2. Motion Module for Cadence Estimation

The motion module placed on the smart facemask was used to assess the ability to estimate the cycling cadence from the accelerations at the level of the head when compared to the cycling cadence extracted from the cycle-ergometer. The idea is that the y-axis (i.e., lateral axis as in Figure 1) of the accelerometer has a cyclical pattern at a frequency resembling that of cycling with a peak local maximum for each pedal stroke of the same foot (defined as an event). Indeed, it is conceivable that the cyclist tends to slightly move his head according to changes in weight distribution during pedalling.

The y-axis accelerations of the motion module were analyzed in the MATLAB environment. Data collected with the cycle-ergometer (reference cadence values) and the motion module were synchronized and split into RIFT traces and HIIT traces. Then, two main steps were performed on the motion module signal to extract the cycling cadence:

- Signal filtering;
- Event-by-event extraction in the time domain.

The signal filtering stage involves the implementation of a first-order BPF with a lower cut-off frequency of 0.01 Hz and a higher cut-off frequency of 3 Hz to better emphasize the contribution of head movement due to the cycling activity. The event-by-event extraction in the time domain consists of selecting the maximum peaks in the signal and computing their distance in time. This corresponds to the period (i.e., $T_{cadence}$) between a head movement between one pedalling cycle and the following one. We then computed the cycling cadence as 60Tcadence expressed in revolutions per minute (rpm). Finally, we applied a Hampel filter to detect and remove outliers, defined as the samples that differ from the median by more than three standard deviations.

**Figure 8:**
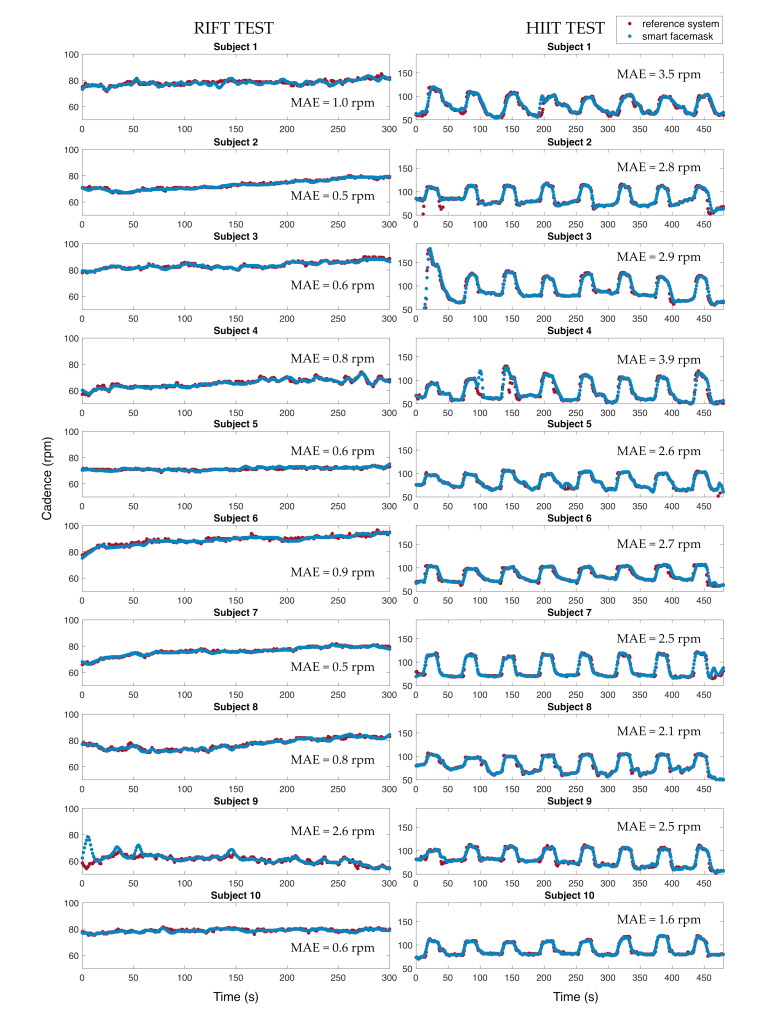
Comparison between the cadence values extracted from the motion module and the reference values extracted from the ergometer for the RIFT test (**left** panels) and the HIIT test (**right** panels).

Cadence values extracted from the motion module were then compared with those extracted from the cycle-ergometer, used as reference ones. To compare the results, the cadence values extracted from the reference cycle-ergometer and our device were resampled at 1 Hz to have one cadence value per second for both devices. A graphical comparison between the cadence values extracted from the two devices for all subjects is shown in Figure 8. In addition, the MAE value for each subject was calculated as shown in Equation (3).

## 6. Outdoor Tests

On a subset of the participants (six), we carried out a second testing session to assess the feasibility of using our system in an outdoor setting with an external temperature (T_E_) higher than 32 °C, and thus closer to T_Exp_ compared to the indoor setting (see Figure 6B). No reference system was used in these tests performed outdoors because the stationary metabolic cart we used could not be transported outside the laboratory. However, this limitation did not prevent us from testing the feasibility of measuring *f*_R_ with the smart facemask, as detailed below.

**Figure 9:**
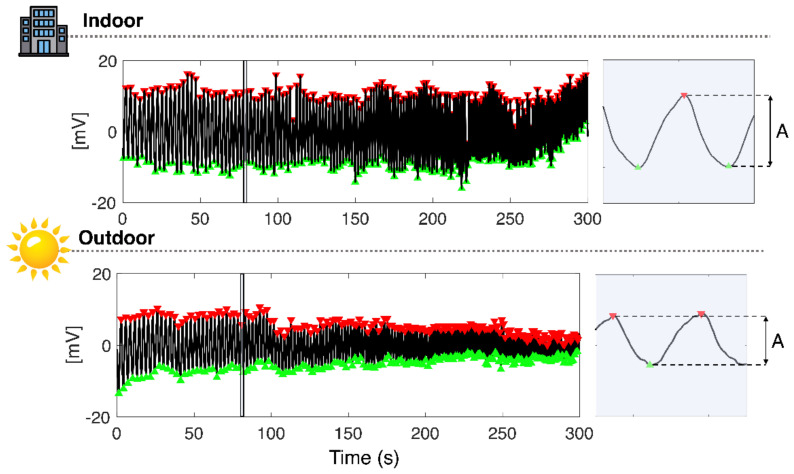
Example of amplitude (A) calculation for one participant in both indoor and outdoor tests. The blue area is the zoom related to one breath.

To investigate the influence of T_E_ on the facemask output signal, we first assessed the amplitude of the signal (A) recorded indoors and outdoors during the RIFT. Per each trial, A was calculated as in the following formula:

(5) $$A\;\left[mV\right]=\frac{1}{N}\overset{N}{\underset{i=1}{\sum }}\left(max_{i}-min_{i}\right),$$

where $max_{i}$. and $min_{i}$ are the maximum (red triangles in Figure 9) and minimum (green triangles) voltage values of the *i*-th breath, respectively. Figure 9 shows the amplitude of the data collected from one volunteer during indoor and outdoor tests.

Table 3 reports the average and standard deviation values of the temperature (T_average_) extracted from the T_E_ signal and the A values for both the indoor and outdoor tests. Despite the A being substantially higher in the indoor test than in the outdoor test, the quality of the temperature respiratory signal was preserved even in the outdoor setting, as shown in Figure 9.

**Figure 10:**
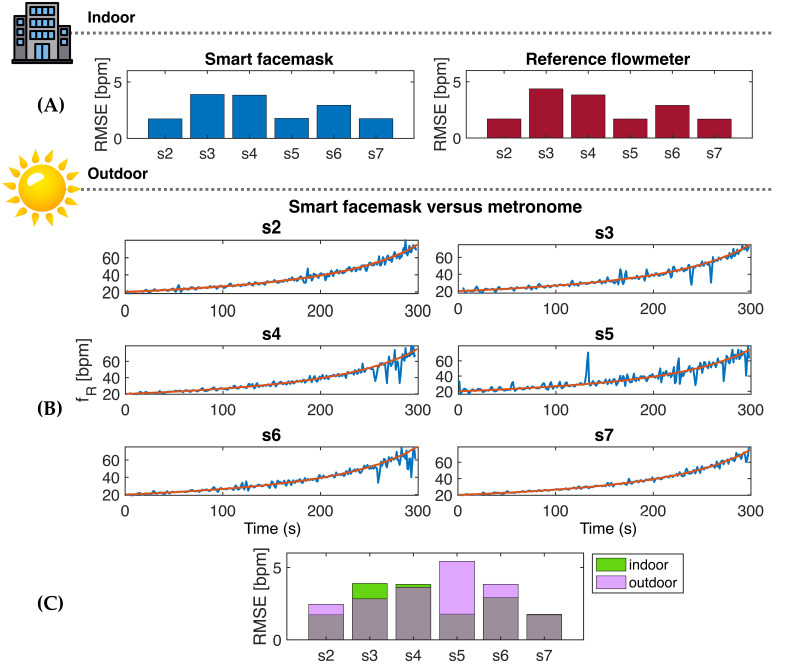
(**A**) RMSE values obtained from the smart facemask and the reference flowmeter when compared to the set *f_R_* values provided by the metronome in indoor tests. (**B**) Time series of the *f*_R_ paced by the metronome (in red) and the *f*_R_ calculated from the facemask (in blue) for each participant during the outdoor test. (**A**,**C**) RMSE values obtained in indoor (green) and outdoor (magenta) tests considering the *f*_R_ calculated from the facemask vs. the *f*_R_ provided by the metronome.

Given the absence of the reference system in the outdoor test, a further assessment of the quality of the temperature signal was made by evaluating the *f*_R_ response during RIFT, where participants were asked to follow the *f*_R_ paced by a metronome. Since participants may commit errors when attempting to follow the pace set by the metronome, we considered this error by calculating an index estimating the ability of the athlete to perform the breathing task. This was made by means of the RMSE value, which was computed during the indoor test, where both the reference signal ϕ and the temperature respiratory signal T_exp_ were recorded. Specifically, we calculated two RMSE values per each volunteer: the first considering the *f*_R_ values provided by the reference system vs. the *f*_R_ values imposed by the metronome, and the second considering the *f*_R_ values provided by the facemask vs. the *f*_R_ values imposed by the metronome. Very similar RMSE values were observed for the facemask and the reference system (see Figure 10A), which outlines that the between-participant differences in RMSE observed were mainly due to errors committed when performing the breathing task rather than representing measurement errors of the facemask. This consideration is important for a correct interpretation of the RIFT data collected in the outdoor setting.

**Figure 11:**
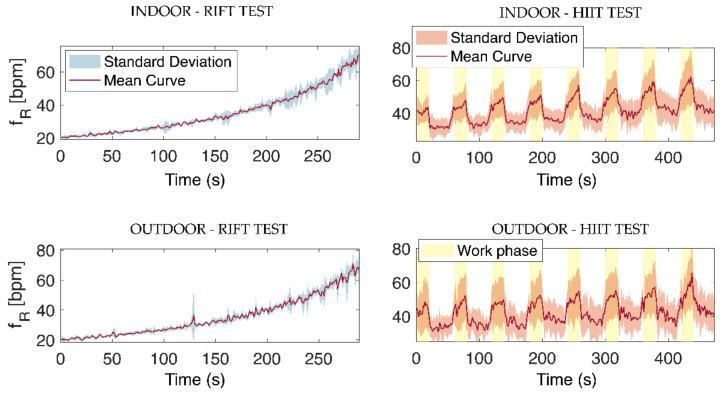
*f_R_* time series from the facemask during the RIFT test (**left** panels) and HIIT test (**right** panels) reported as mean ± standard deviation values. The trends shown were obtained by averaging the responses of all the subjects during both indoor and outdoor tests. The work phase of the HIIT tests is highlighted in yellow.

The RMSE value of data collected during the outdoor test was then calculated for each participant using the *f*_R_ values provided by the smart facemask (blue line, Figure 10B) vs. the *f*_R_ values imposed by the metronome (red line). Similar RMSE values were generally found for the same participant in the indoor and outdoor tests, which further suggests that the small discrepancy observed between the *f*_R_ values of the facemask and those set by the metronome was largely due to errors made by the participants when performing the breathing task. Notably, even without deducting the error made by participants from the RMSE computation, the *f*_R_ response observed during RIFT closely resembled the *f*_R_ time course set by the metronome, as depicted in Figure 10 and Figure 11. The *f*_R_ time course observed during HIIT both during indoor and outdoor tests (see Figure 11) further supports the quality of the data collected from the smart facemask, as the observed response is in line with the response reported in the literature during similar HIIT tests performed in the cycling modality [2,8,16].

## 7. Discussions

The purpose of this study was to test the validity of a wearable smart facemask measuring *f*_R_ during cycling exercise. The custom-made system was developed to suit the requirements of athletes and exercise practitioners, both in terms of wearability and quality of the respiratory signal. When compared to the reference system, we found a very good performance of the smart facemask in measuring *f*_R_, both during progressive increases in *f*_R_ paced by a metronome and during HIIT. Precision and accuracy were very good, and the MAE was always lower than 2 bpm, even when the validity of the *f*_R_ measurement was assessed at a breath-by-breath level. Moreover, when the validity was assessed on *f*_R_ values averaged every 30 s, the MAPE was always lower than 1%. We found that the quality of the respiratory temperature signal was preserved even when tests were performed outdoors at temperatures above 30 °C, despite a substantial reduction in the amplitude of the signal. Both the indoor and outdoor HIIT tests showed the characteristic fast response of *f*_R_ to the alternation of work and recovery (see Figure 11), which makes *f*_R_ particularly suitable for monitoring high-intensity exercise [2,7,8,16]. These findings have important implications for both research and exercise management in different populations.

The very low errors found for the proposed facemask have rarely been reported when testing the validity of other wearable devices during exercise. Not only is our solution superior to recent attempts made in extracting *f*_R_ from cardiac signals [28,29], but it also appears to overperform commercial and custom-made wearable devices integrating strain sensors. For instance, substantially higher error values than those observed in our study were found when assessing the validity of the Zephyr^TM^ BioHarness^TM^ chest strap of the Hexoskin^®^ smart shirt and of the capaciflector sensors tested by Hayward et al. [19,20,23,24,25,26]. Notably, the performance of our facemask is even superior to that of a custom-made wearable device integrating a differential pressure sensor previously tested in similar exercise conditions and using similar analyses [30]. During HIIT, the previous study found average MAPE values of 4.03% and 1.77% when computed breath-by-breath or on 30 s windows, respectively [30], while we found average values of 1.64% and 0.23%, respectively, in this study. The very good performance of our smart facemask is not surprising if we consider that the good quality of the respiratory temperature signal has long been exploited in the field of exercise physiology for the evaluation of the locomotor-respiratory coupling phenomenon [32,33,34,35,36,37]. Indeed, its assessment requires the accurate measurement of *f*_R_ and its subcomponents at a breath-by-breath level. However, the good metrological characteristics of the temperature sensors have not so far been effectively used for developing wearable sensors measuring *f*_R_ during exercise, and our study makes an important step in this direction.

The performance of the smart facemask presented herein is substantially better than that of a preliminary system that we tested in our laboratory [31], and several factors explain the improvements made in the quality of the temperature respiratory signal. The preliminary system had a temperature sensor embedded into the electronic board and had thus been located outside of a commercial facemask used for exercise testing. That system was prone to artefacts caused by wind and had only been preliminarily tested in the laboratory [31]. Conversely, the herein-presented smart facemask was developed with account taken of the needs of both indoor and outdoor exercise testing. Hence, the temperature sensor was not embedded into the electronic board but was conveniently located in the facemask and integrated into a spoon-shaped housing system allowing the sensor to be directly exposed to nose and mouth airflow and unexposed to wind. As a result, our smart facemask resulted in about 2–4 times lower MAPE values compared to the previously tested preliminary system [31].

Before discussing the important implications of our findings, we acknowledge the limitation of monitoring exercise with a facemask, albeit wearable and light, as this prevents the possibility for athletes and exercise practitioners from wearing it daily. Indeed, the devices that athletes commonly wear on a daily basis are less obtrusive (e.g., heart rate straps, global positioning systems, etc.). On the other hand, the assessment of athletes’ ventilatory responses has so far been performed with devices requiring the use of a facemask (mostly in the laboratory), and hence athletes are used to wearing a facemask for exercise testing. Our smart facemask may play an important role in the attempt to move respiratory monitoring from the laboratory to the field. Indeed, not all the attempts made so far by companies or researchers to use unobtrusive wearable devices for measuring *f*_R_ have resulted in valuable information provided to the athlete or the exercise practitioner. For instance, when using some analyses/sensors for processing/recording the cardiac signal to extract *f*_R_ [28,29], the *f*_R_ measurement error may not be negligible. In fact, this issue may also apply to a variety of other sensors and devices because it is common practice to perform validation studies in the laboratory and assume that the devices under validation would exhibit similar performance in the field. However, some researchers have encouraged the verification of this assumption because different exercise protocols, modalities, and environmental factors may affect the quality of the respiratory signal [28,29]. Hence, our facemask meets the current need of the sports science community to have a wearable, light, affordable, accurate, and precise device that can be used to test the validity of *f*_R_ measurements made by other wearable devices in the field. This implies that our facemask can also be used for applied research performed in the field, thus facilitating our understanding of how *f*_R_ responds and is regulated in applied exercise settings.

In an attempt to extract valuable information from the *f*_R_ response, it is important to monitor other variables to understand the context leading to the observed response. The movement module of the smart facemask is not only suitable for recognizing if the user is cycling or not, but it also appears to provide pedalling cadence values with relatively low errors. Indeed, we observed MAE values always lower than 4 rpm even during HIIT (the values ranged from 1.9 to 3.9 rpm), where pedalling cadence values higher than 100 rpm were found in all the participants. This is important because pedalling cadence affects *f*_R_, although we have found that *f*_R_ does not change in proportion to pedalling cadence as this relationship is moderated by exercise intensity [47]. Indeed, it is more likely to observe consistent changes in pedalling cadence and *f*_R_ during low-to-moderate exercise than during high-intensity exercise [47]. Nevertheless, the fact that pedalling cadence may confound the association between *f*_R_ and perceived exertion (especially at low intensities) [47] outlines the need to measure pedalling cadence alongside *f*_R_, and our smart facemask appears to be suitable for this purpose.

While we do not advise the use of the herein-presented facemask for monitoring athletes on a daily basis, the device can be used for a variety of other applications requiring accurate *f*_R_ measurements. Performance assessment is fundamental for managing the training process, and our device is suitable for use during both maximal and submaximal tests [31]. Indeed, the fact that the *f*_R_ time course is sensitive to changes in exercise capacity makes respiratory monitoring suitable for gaining insights into performance adaptations [10]. There is also an emerging interest in the voluntary modulation of the breathing pattern during exercise [48]. Although further research is needed before any suggestion can be made on the acute or chronic use of breathing strategies during exercise, our smart facemask may support research in this area and facilitate the practice of breathing strategies. Furthermore, the accurate measurement of *f*_R_ has the potential to refine exercise prescription and monitoring in patients. Indeed, all the patients experiencing a reduction in exercise capacity, not only the patients affected by pulmonary diseases, have an anticipated increase in *f*_R_ compared to healthy individuals [10], and the sensitivity of *f*_R_ to changes in exercise tolerance may prove useful for monitoring patients.

While the characteristics of our smart facemask make it suitable for *f*_R_ monitoring in a variety of sporting activities and populations, our findings may not be generalized to conditions different from those tested in the present study until further research is performed. For instance, we have not tested female participants, although there is no reason to assume that the temperature respiratory waveform is substantially different between males and females. Hence, while gender differences should be considered when assessing wearable devices recording respiratory-induced movements, this issue is negligible for devices measuring airflow temperature. Unlike the respiratory waveform recorded with strain sensors, the temperature respiratory waveform is neither substantially affected by interindividual differences in anthropometric characteristics nor movement artefacts, and this contributes to explaining why we found similar errors of measurement between different participants. This suggests that our findings can be generalized to healthy, trained male individuals performing cycling exercise despite the relatively low sample size tested. On the other hand, we cannot exclude that the use of a facemask may affect the breathing pattern, and interest in this issue has been further stimulated by recent research exploring the physiological effects of the protective masks used for contrasting the spread of the COVID-19 virus. The systematic review by Zheng et al. [49] suggests that the use of facemasks during exercise does not substantially affect *f*_R_, which encourages the use of our facemask that was specifically designed to minimize respiratory resistance while making the temperature sensor unexposed to wind. However, further research is required to specifically test the effects of our facemask on *f*_R_ and exercise performance.

## 8. Conclusions

This study shows the very good performance of a wearable smart facemask measuring *f*_R_ during cycling exercise with a temperature sensor. The quality of the temperature respiratory signal was preserved even in outdoor scenarios with external temperatures above 30 °C. This device appears to be generally superior to other custom-made and wearable devices developed for similar purposes and can thus be used for the validation of other devices in applied settings. Other applications include exercise testing and management in different populations, ranging from athletes to patients. While the convenient design of the smart facemask makes it suitable for monitoring a variety of other sports and exercise modalities beyond cycling, further research is needed to address its performance in conditions different from those assessed in this study.

## Figures and Tables

**Table 1:** Breath-by-breath analysis results: (**A**) RIFT test and (**B**) HIIT test.

(A) Breath-by-Breath Analysis—Rift Test					(B) Breath-by-Breath Analysis—Hiit Test				
Cyclist	#Breathing Events	Bias [MOD ± LOAs] [bpm]	MAE [bpm]	MAPE [%]	Cyclist	#Breathing Events	Bias [MOD ± LOAs] [bpm]	MAE [bpm]	MAPE [%]
1	173	−0.02 ± 2.57	0.89	2.30	1	320	−0.04 ± 3.85	1.00	2.54
2	177	−0.01 ± 2.50	0.93	2.48	2	371	−0.01 ± 1.68	0.60	1.29
3	174	−0.23 ± 4.84	1.02	2.60	3	416	0.00 ± 2.00	0.68	1.22
4	175	−0.01 ± 2.92	1.10	2.74	4	290	−0.03 ± 2.26	0.85	2.33
5	179	−0.03 ± 4.62	1.75	4.50	5	245	−0.01 ± 2.31	0.77	2.58
6	174	−0.02 ± 2.58	0.75	1.82	6	283	0.00 ± 1.40	0.50	1.43
7	179	−0.01 ± 2.08	0.66	1.72	7	306	−0.04 ± 1.32	0.48	1.13
8	179	−0.02 ± 2.68	1.05	2.74	8	331	−0.03 ± 1.85	0.59	1.48
9	174	−0.11 ± 2.87	0.98	2.34	9	362	−0.06 ± 3.43	0.75	1.67
10	174	−0.02 ± 2.52	0.84	2.31	10	377	0.00 ± 2.25	0.66	1.28
**Overall**	**1758**	**−0.05 ± 3.37**	**1.00**	**2.56**	**Overall**	**3301**	**−0.02 ± 2.37**	**0.69**	**1.64**

**Table 2:** 30 s window analysis results: (**A**) RIFT test and (**B**) HIIT test.

(A) 30 s—Window Analysis—Rift Test					(B) 30 s—Window Analysis—Hiit Test				
Cyclist	#Windows	Bias [MOD ± LOAs] [bpm]	MAE [bpm]	MAPE[%]	Cyclist	#Windows	Bias [MOD ± LOAs] [bpm]	MAE [bpm]	MAPE [%]
1	10	−0.03 ± 0.31	0.10	0.28	1	16	−0.13 ± 0.28	0.14	0.38
2	10	−0.01 ± 0.26	0.10	0.34	2	16	−0.01 ± 0.14	0.06	0.13
3	10	−0.10 ± 0.42	0.17	0.58	3	16	−0.06 ± 0.43	0.09	0.16
4	10	−0.04 ± 0.29	0.10	0.27	4	16	−0.03 ± 0.15	0.07	0.19
5	10	−0.02 ± 0.54	0.18	0.62	5	16	0.00 ± 0.20	0.08	0.27
6	10	−0.05 ± 0.12	0.06	0.20	6	16	0.00 ± 0.10	0.04	0.13
7	10	−0.02 ± 0.15	0.06	0.19	7	16	0.04 ± 0.53	0.12	0.30
8	10	−0.06 ± 0.22	0.09	0.27	8	16	−0.02 ± 0.13	0.06	0.15
9	10	−0.04 ± 1.16	0.30	0.75	9	16	0.01 ± 0.78	0.19	0.39
10	10	0.00 ± 0.19	0.07	0.25	10	16	−0.07 ± 0.28	0.09	0.19
**Overall**	**100**	**−0.02 ± 0.45**	**0.12**	**0.37**	**Overall**	**160**	**−0.03 ± 0.37**	**0.09**	**0.23**

**Table 3:** Average temperature (Taverage) and amplitude (A) expressed as mean ± standard deviation for both indoor and outdoor tests.

Cyclist	T_average_ Indoor [°C]	A [mV] Indoor	T_average_ Outdoor [°C]	A [mV] Outdoor
2	25.7 ± 0.1	10.3 ± 3.6	34.6 ± 0.3	2.8 ± 1.6
3	26.2 ± 0.1	12.2 ± 4.2	33.4 ± 0.6	6.3 ± 2.8
4	25.8 ± 0.1	7.4 ± 2.4	31.2 ± 0.2	4.0 ± 1.8
5	25.8 ± 0.1	5.9 ± 2.2	32.9 ± 0.3	2.5 ± 1.3
6	26.9 ± 0.1	11.3 ± 4.1	30.8 ± 0.5	3.0± 1.6
7	26.0 ± 0.2	18.5 ± 3.7	31.0 ± 0.3	9.3 ± 4.0

## Data Availability

The data presented in this study are available on request from the corresponding author. The data are not publicly available due to privacy reasons.
